# Climate variability differentially impacts thermal fitness traits in three coprophagic beetle species

**DOI:** 10.1371/journal.pone.0198610

**Published:** 2018-06-06

**Authors:** Casper Nyamukondiwa, Frank Chidawanyika, Honest Machekano, Reyard Mutamiswa, Bryony Sands, Neludo Mgidiswa, Richard Wall

**Affiliations:** 1 Department of Biological Sciences and Biotechnology, Botswana International University of Science and Technology (BIUST), Palapye, Botswana; 2 Agricultural Research Council, Plant Protection Research Institute, Weeds Division, Hilton, South Africa; 3 School of Lifesciences, University of KwaZulu-Natal, Pietermaritzburg, South Africa; 4 School of Biological Sciences, University of Bristol, United Kingdom; Museum National d'Histoire Naturelle, FRANCE

## Abstract

While the impacts of extreme and rising mean temperatures are well documented, increased thermal variability associated with climate change may also threaten ectotherm fitness and survival, but remains poorly explored. Using three wild collected coprophagic species *Copris elphenor*, *Metacatharsius opacus* and *Scarabaeus zambezianus*, we explored the effects of thermal amplitude around the mean on thermal tolerance. Using standardized protocols, we measured traits of high- (critical thermal maxima [CT_max_] and heat knockdown time [HKDT]) and -low temperature tolerance (critical thermal minima [CT_min_], chill coma recovery time [CCRT] and supercooling points [SCPs]) following variable temperature pulses (δ0, δ3, δ6 and δ9°C) around the mean (27°C). Our results show that increased temperature variability may offset basal and plastic responses to temperature and differs across species and metrics tested. Furthermore, we also show differential effects of body mass, body water content (BWC) and body lipid content (BLC) on traits of thermal tolerance. For example, body mass significantly influenced *C*. *elphenor* and *S*. *zambezianus* CT_max_ and *S*. *zambezianus* HKDT but not CT_min_ and CCRT. BWC significantly affected *M*. *opacus* and *C*. *elphenor* CT_max_ and in only *M*. *opacus* HKDT, CT_min_ and CCRT. Similarly, BLC only had a significant effect for *M opacus* CT_min._ These results suggest differential and species dependent effects of climate variability of thermal fitness traits. It is therefore likely that the ecological services provided by these species may be constrained in the face of climate change. This implies that, to develop more realistic predictions for the effects of climate change on insect biodiversity and ecosystem function, thermal variability is a significant determinant.

## Introduction

The frequency and magnitude of extreme thermal events, including deviations from the mean environmental temperatures, is increasing due to climate change [1, 2]. These changes in both seasonal and diurnal temperature fluctuations can pose significant physiological challenges for many species [3,4], with ecological consequences that may have direct implications for biodiversity conservation [5–8]. For ectothermic arthropods, the ability to withstand both acute and chronic thermal variability is even more critical, as body temperature is largely dependent on ambient temperature [9–11]. Deviations from optimal ranges not only affect physiological processes (e.g. metabolic rates), but also key activities such as locomotion [12,13], mating, and reproductive success [14,15] and ultimately survival [16,17]. As such, the ability of organisms to operate within varying magnitudes of temperature fluctuation may be a critical trait in the face of climate change.

Phenotypic plasticity is an adaptive response associated with thermal history, that can reduce potential vulnerability to thermal extremes [8,18] both within and across generations [19,20]. For example, rapid hardening, which is a process in which an acute exposure to a sub-lethal temperature can improve subsequent performance and survival in otherwise lethal thermal conditions [21–24]. Acclimation through a chronic prior exposure to conditions similar to a future stressful environment, can improve survival and performance [25,26]. This acclimation can be realized in periods ranging from a few days to even months [11,27,28]. In many cases, such phenotypic plasticity leads only to transient physiological and biochemical responses [29–31], nevertheless, it still optimizes key life history traits during otherwise deleterious environmental conditions. Under longer timescales plasticity can lead to local genetic adaptation through changes in allele frequency [32–33].

Whilst phenotypic plasticity in response to thermal variability is pervasive in nature, several factors mediate its occurrence among different insect species. Diet quality and starvation [34–36,16], ontogeny [37,38], body size [39,40] and age [41,16] are all known to influence biochemical processes and subsequent thermal performance or survival. Indeed, strong links have been reported between body size and physiological traits such as body water and lipid content [9,42,40] with concomitant impact on climatic stress resistance [43–45,36,46]. Apart from insect physiological attributes, there is increasing evidence of how the pattern of thermal variability can also influence both constitutive (basal) and acquired (plastic) thermal responses. For example, rapid thermal variability, typical of that occurring under climate change scenarios, may exacerbate the impact of warming [47–53].

The effects of thermal history pose challenges for studies of the effects of thermal variability, because most use laboratory insect populations, typically reared under constant temperatures [54–56]. Furthermore, most studies focus on changes in mean temperatures excluding the magnitude of thermal fluctuations [57]. Thus, more robust studies on thermal variability around the mean rather than variations of the mean itself warrant investigation, in order to better understand the likely impacts of climate change on insect fitness (see [7]). As a result, here we use ecologically relevant standardised protocols (see [58,59]) to determine climate stress resistance among field-collected coprophagic beetles.

Coprophagic beetles (Coleoptera: Scarabaeidae) perform important ecosystem services such as secondary seed dispersal and nutrient cycling through degradation and burying of dung [60–62]. Furthermore, dung colonizing beetles serve as important biological control agents of intestinal parasites [63] and several pest flies [64,65] through rapid removal of the dung in the field. Due to their high diversity, abundance and important ecosystem functions, dung beetles have become a focal taxon for various ecological studies including impacts of habitat degradation [61,66–69]. The functional diversity of dung beetles has also been reported as an important buffer for such ecosystem services in disturbed agro-ecosystems [61]. In addition, dung beetles exhibit different adult body sizes across species and losses of some ecological functions have already been attributed to poor assemblages of large sized beetles [69]. Even though the factors leading to impoverished assemblages of large sized beetles are not well understood, we hypothesize that body size differentially influences vulnerability to environmental variables, as reported in other insect taxa (e.g. [43,44,40]). How changes in mean temperature and variability affect static and plastic thermal functional traits are important for elucidating insect physiological responses [7,70]. Here we use ecologically relevant protocols [58,59] to determine climate stress resistance among field-collected coprophagic beetles. Thus we test the impact of thermal variability on overall fitness, specifically, the ability of two paracoprid (tunneller) species: *Copris elphenor* Klug, 1855, *Metacatharsius opacus* Waterhouse, 1981 and one telocoprid (ball-rolling) *Scarabaeus zambezianus* Péringuey, 1901 to tolerate low and high temperature stress across a gradient of body mass using field collected specimens. The species to conduct the study were collected from field sites in Botswana, and they were the representatives most abundant that were active at the time of sampling.

## Materials and methods

### Sampling site and study animal preparation

A permit was granted from the Ministry of Environment, Wildlife and tourism Botswana [number EWT 8/36/4 XXXIII (18)] to collect experimental organisms. Dung beetles were sampled directly from the field in Khumaga Village (S20.46801; E24. 51491; 918 m.a.s.l), Central District, Botswana–a semi-arid region with Kalahari sand soils. Collections were made at the onset of the rainy season (January and February 2017), the peak period of adult emergence, to increase the likelihood of collecting beetles sharing a similar thermal history. Khumaga is characterized by abundant wildlife and domestic animals which supports high coprophagic species diversity. The area has typical average annual rainfall of 34.4 mm, most of which is recorded in January/February, and average temperatures of 23.6°C (Botswana Meteorological Services). The minimum and maximum temperatures for the two sampling months was 18.5 and 46°**C** respectively. Dung beetles were collected using pitfall traps baited with fresh cow dung. The traps comprised several ~8000 ml plastic buckets that were covered with a wire mesh (~5cm diameter holes) and then buried so that they were flush with the ground. The traps were equipped with a plastic rain guard that was placed 20 cm above the traps to prevent flooding. Every evening, fresh dung was placed on the wire mesh to attract foraging beetles to land and fall into the trap. A UV light bulb was also mounted on each trapping station as a light source to attract the beetles. Beetle trapping was undertaken over 12 h periods (18:00–06:00 h) and the beetles were then collected from the traps each morning. The beetles were immediately sorted according to species, based on morphological features described by Davis et al [71], and stored in insulated Styrofoam containers that had soil and fresh dung for feeding *ad libtum* during transportation to the laboratory. During the sampling period, the prevailing temperature was recorded using iButtons (model DS1923, Maxim, Sunnyvale, CA, USA). The iButtons were set within freshly deposited dung and at ground level where they were lightly covered with soil to determine potential differences in dung and ground temperature.

### Thermal variability treatments

**Fig 1:**
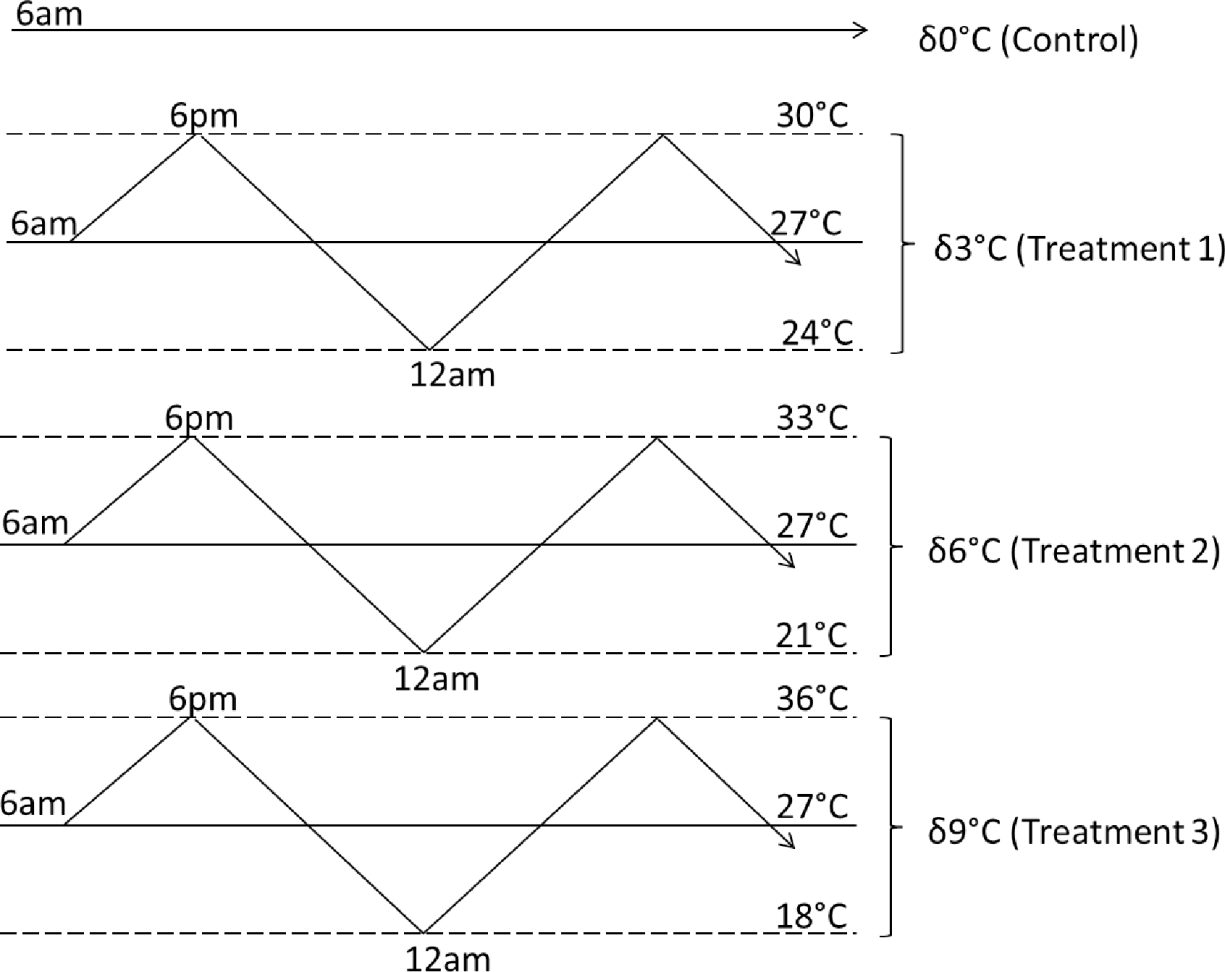
Schematic representation of acclimation treatments to variable temperatures at different amplitudes oscillating around an ambient mean temperature of 27°C. The control (δ0°C) was kept at benign 27°C.

In the laboratory, insects were acclimated in climate chambers (HPP 260, Memmert GmbH + Co.KG, Germany) at 65 ± 10% relative humidity (RH) under 14L:10D at three different fluctuating thermal regimes (δ3, δ6 and δ9°C) around 27°C (Fig 1) for five days. Control beetles were placed in a chamber at constant 27°C (δ0), 65 ± 10% RH and 14L:10D (Fig 1). Temperature and RH in each cage was verified using iButton data loggers.

### Effects of thermal variability on high temperature plasticity

#### Critical thermal maxima (CT_max_)

Critical thermal maximum was measured for insects from the three treatments described above using the protocol outlined by Nyamukondiwa and Terblanche [41] and Mudavanhu et al [72]. First, ten mixed sex adult beetles were numbered and individually weighed using an electronic microbalance (RADWAG^®^ Wagi Elektroczne, Model AS220.R2) for each of the three species and temperature treatments. This was done following 12 h starvation to allow for the clearance of gut contents and reduce the possible confounding effects on temperature tolerance [41]. Thereafter, the beetles were placed in a series of insulated double-jacketed chambers (‘organ pipes’) connected to a programmable water bath (Lauda Eco Gold, Lauda DR.R. Wobser GMBH and Co. KG, Germany) filled with 1:1 water: propylene glycol [9]. The beetles were then first given 10 min to equilibrate at 27°C (equivalent to the rearing benign temperature) before increasing the temperature (CT_max_) at a rate of 0.25°C min^−1^. This was repeated twice to give a sample size of *n* = 20 for each species per each treatment. A thermocouple (type K 36 SWG) connected to a digital thermometer (53/54IIB, Fluke Cooperation, USA) was inserted into the control chamber to monitor chamber temperatures. Individual beetle body temperature was assumed to be in equilibrium with the chamber temperature under the experimental conditions [58,72]. The CT_max_ was defined as the temperature at which each individual insect lost coordinated muscle function, consequently losing the ability to self-right or respond to mild stimuli (like prodding with a soft thermally inert camel-hair brush) [19]. After recording, individual beetles were returned to the labeled vials fitted with gauzed lids and then placed in khaki envelopes which were placed in Memmert oven (UF 160, Memmert GmbH + Co.KG, Germany) set at 60°C for 48 h. Individual beetle weight was immediately recorded thereafter and subtracted from initial body weight to determine body water content following methods by Weldon et al. [73]. Body lipid content for *M*. *opacus* and *C*. *elphenor* was investigated and correlated with CT_max_, following modifications from Lease & Wolf [42]. Individual adult beetles were weighed in pre-weighed 50ml Eppendorf tubes, then dried in an oven (UF160, Memmert, Germany) at 60°C for 48 h. Following drying, the flies were weighed on a RADWAG microbalance (model AS 220.R2, Poland; precision 0.001mg). Thereafter, 1.5ml of diethyl ether was added to each tube and then gently agitated at 250 rpm for 24h at 37°C using orbital shaker. The diethyl ether was then removed from the tubes, and the flies dried again at 60°C for 24h, before reweighing. The lipid content of each beetle was calculated by subtracting the lipid free dry mass from the initial beetle dry mass [42].

#### Heat knockdown time (HKDT)

After exposure to the thermal variability treatments described in the previous section, HKDTs for the three beetle species were assayed as outlined by Weldon et al [73]. Ten beetles were weighed individually and placed in numbered 2 ml Eppendorf tubes (*M*. *opacus*) and 30ml polypropylene vials (*C*. *elphenor and S*. *zambezianus*) and placed in a climate chamber set at 48 ± 0.5°C (65 ± 10% RH) connected to a camera (HD Covert Network Camera, DS-2CD6412FWD-20, Hikvision Digital Technology Co., Ltd, China) linked to a computer. This was repeated two times to yield sample sizes of *n* = 20 for each species and temperature treatment. All observations from the climate chamber were recorded from a video recording on the computer screen. In this study, HKDT was defined as the time (in minutes) at which an individual beetle lost activity due to heat in the climate chamber as observed from the camera.

### Effects of thermal variability on low temperature plasticity

#### Critical thermal minima (CT_min_)

For comparative low temperature tolerance of *C*. *elphenor*, *M*. *opacus*, and *S*. *zambezianus* across all treatments, critical thermal minima (CT_min_) were measured using standardized protocol as outlined by Nyamukondiwa and Terblanche [41]; Mudavanhu et al [72]. As in the CT_max_ experiments, ten replicate beetles were weighed individually and placed in numbered ‘organ pipes’ connected to a programmable water bath before decreasing the temperature at a rate of 0.25°C/min until their CT_min_ were recorded. This was also repeated twice to yield sample sizes of *n* = 20 per species. In this study, CT_min_ was regarded as the temperature at which each individual insect lost coordinated muscle function, consequently losing the ability to respond to mild stimuli (e.g. gentle prodding). The BLC of *M*. *opacus* and *C*. *elphenor* was determined following previous protocol [42], and its relationship with CT_min_ was calculated.

#### Chill Coma Recovery Time (CCRT)

Chill Coma Recovery Time experiments were assayed as outlined by Weldon et al [73]. As in the CT_min_ experiments, individual weights of *C*. *elphenor*, *M*. *opacus*, and *S*. *zambezianus* were first recorded as described. After weighing, ten beetles (from each species) were individually placed in 30ml polypropylene vials with gauzed screw-cap lids and then loaded into a large zip-lock bag which was submerged into a water bath (Systronix, Scientific, South Africa) filled with 1:1 water: propylene glycol set at 0°C for 1 h. This temperature by time treatment has been previously reported to elicit chill-coma in other insect taxa (see [73,74]). Following 1 h treatment at chill-coma temperature, the vials were immediately removed from the water bath and placed in a climate chamber set at 27±1°C, 65±10% RH for recovery. The chamber was connected to a video recording camera which was linked to a computer for recording observations. This was repeated two times to yield sample sizes of *n* = 20 per treatment. CCRT was defined as the time (in mins) required for an adult to stand upright on its legs following recovery from chill-coma [75].

#### Supercooling points (SCPs)

Supercooling points for the three beetle species were assayed following the protocol of Nyamukondiwa et al [76]. Sixteen beetles of each species were individually placed into 30 ml polypropylene vials with gauzed screw-cap lids. Each beetle was placed in contact with the tip of a type-T copper-constantan thermocouple (762–1121, Cambridge, UK), inserted through the gauzed lid of the vial and both the beetle and thermocouple were secured in contact by a cotton wool. Thermocouples were connected to one of two 8-channel Picotech TC-08 (Pico Technology, Cambridge, UK) thermocouple interfaces and temperatures were recorded at 1s intervals using PicoLog software for windows (Pico Technology, Cambridge, UK). In all treatments, experiments started by holding individual insects at 15°C for 10 mins (for insects’ temperature equilibration) before decreasing the temperature at a rate of 0.5°C min^-1^ until SCPs were recorded. SCP for each individual was determined as the lowest temperature recorded prior to a spike in temperature associated with the latent heat of crystallization [76].

### Data analysis

Data was analyzed in STATISTICA 13.0 (Statsoft Inc., Tulsa, Oklahoma) and R version 3.3.0 [77]. SCPs, HKDT and CCRT data did not meet the assumptions of ANOVA and thus were analyzed using generalized linear models (GLZ) assuming a Gaussian distribution and a logit link function [27] in R3.3.0 statistical software. CTLs (CT_max_ and CT_min_) met the linear model assumptions of constant variance and normal errors, so the data were analyzed using one-way factorial ANOVA in STATISTICA 13.0; Tukey-Kramer’s *post-hoc* tests were used to separate statistically heterogeneous means. The relationship between traits of thermal tolerance and body mass, body water content (BWC) and BLC were examined using linear regression in STATISTICA. Differences in environmental ambient temperature and inside the dung were compared graphically using Origin 8 software (OriginLab Corporation^®^, Northampton, Massachusetts, USA).

## Results

### Effects of thermal variability on high temperature plasticity

**Fig 2:**
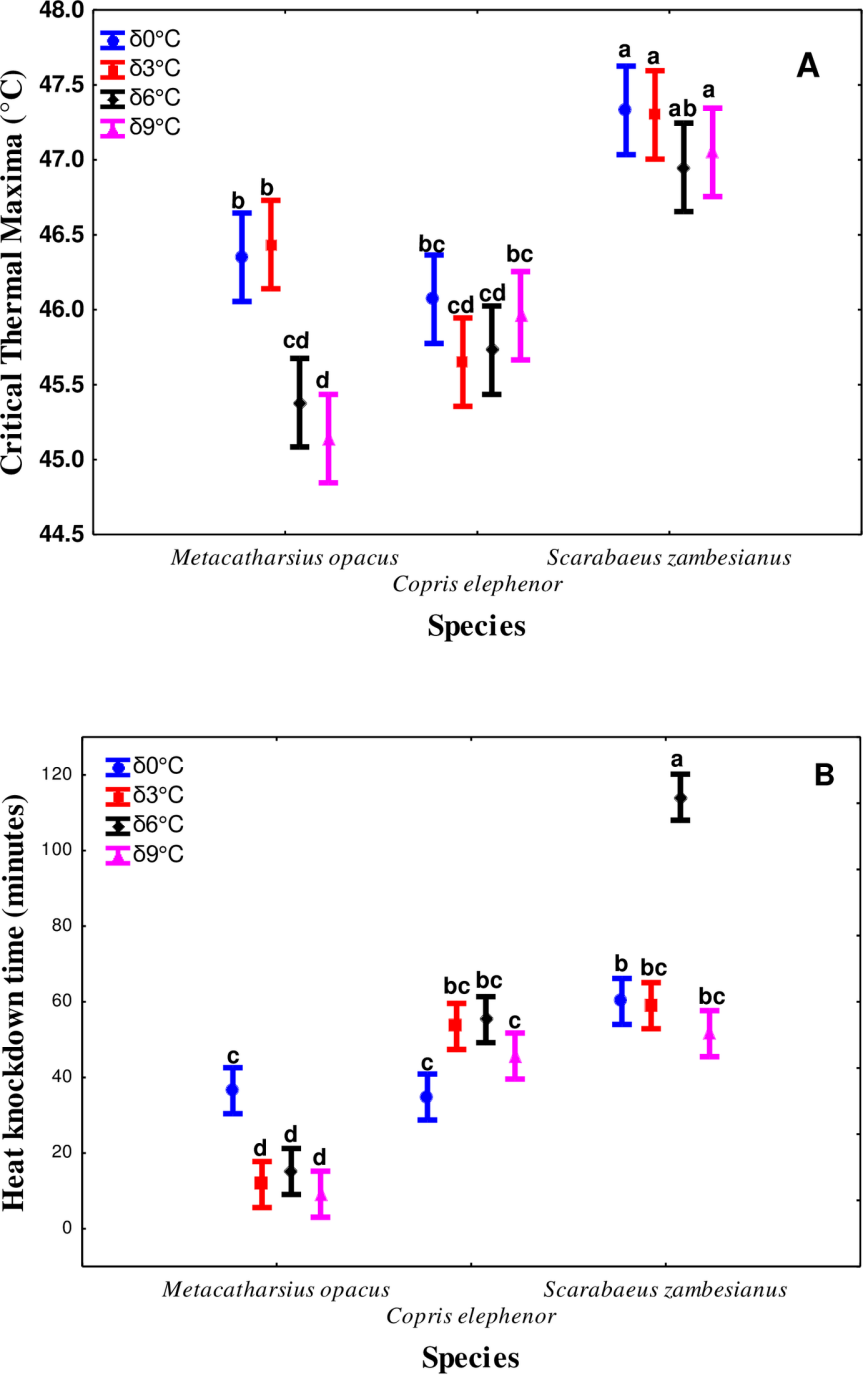
Effect of temperature fluctuation (acclimation at temperatures pulsating around a benign of 27°C: 0 = δ0°C (unacclimated), 3 = δ3°C, 6 = δ6°C and 9 = δ9°C) on (A) Critical thermal maxima and (B) Heat knock down time for *M*. *opacus*, *C*. *elphenor* and *S*. *zambesianus*. Vertical bars denote ±95% confidence limits. Means with the same letter are not statistically different.

Thermal variability significantly affected CT_max_ across all species tested (F_3,11_ = 14.7, *P* < 0.001). Higher thermal variability (δ6 and δ9°C) was associated with a significantly lower high temperature tolerance, and this trend was more pronounced in *M*. *opacus* relative to *C*. *elphenor* and *S*. *zambezianus* (Fig 2A). Similarly, there was a significant species (F_2,103_ = 92.7, *P* < 0.001) and species x treatment interaction effect (F_6,6_ = 16.0, *P* < 0.001) for CT_max_. Overall, *S*. *zambezianus* had relatively higher CT_max_ (47.16±0.083°C) compared to *M*. *opacus* (45.83±0.104°C) and *C*. *elphenor* (45.85±0.058°C) across all treatments (Fig 2A).

Furthermore, thermal variability treatments (*χ*^2^ = 118.6, d.f = 3, *P* < 0.001) and species (*χ*^2^ = 592.6.78, d.f = 2, *P* < 0.001) also affected HKDT across all species. The interaction between species and treatment (*χ*^2^ = 219.9, d.f = 6, *P* < 0.001) also influenced HKDT. *Scarabaeus zambezianus* had the highest HKDT indicating enhanced high temperature tolerance (Fig 2B). However, higher thermal variability had contrasting effects on the different species. While increased magnitude of temperature variability generally was associated with higher HKDT in *C*. *elphenor* (51.99±1.830 minutes) and *S*. *zambezianus*, (75.50±4.345 minutes) it was lower in *M*. *opacus* (12.20±0.372 minutes) (Fig 2B).

The relationship between traits of high temperature tolerance versus species body mass had contrasting results (Table 1). Generally, when all species were pooled together, body mass was not significantly related to high temperature tolerance. However, when species were analysed individually, body mass was significantly related to *C*. *elphenor* and *S*. *zambezianus* CT_max_ and *S*. *zambezianus* HKDT (Table 1). The effects of BWC on heat tolerance were more apparent both across and within species (Table 2). Due to limitations in the numbers of beetles available, it was not possible to examine the effect of BLC on all species, and this was only considered for *M*. *opacus* and *C*. *elphenor*. Nevertheless, BLC was not significantly correlated with high temperature tolerance (measured as CT_max_) (Table 3).

**Table 1:** The relationship between traits of high and low temperature tolerance and body mass. Analysis was performed using linear regression in STATISTICA. Dung beetle species body mass were correlated with traits of temperature tolerance independently and then all species combined. *R* represents the coefficient of correlation.

**Traits of high temperature**	**r**	***p***
*Critical Thermal Maxima (CT*_*max*_*)* **vs Body mass**		
All species	0.2211	0.0895
*M*. *opacus*	-0.3639	0.1147
*C*. *elphenor*	-0.4506	0.0462
*S*. *zambesianus*	0.4969	0.0258
*Heat Knockdown Time (HKDT)* **vs Body mass**		
All species	0.0628	0.6338
*M*. *opacus*	0.5598	0.0055
*C*. *elphenor*	0.0394	0.8689
*S*. *zambesianus*	-0.6497	0.0019
**Traits of low temperature**		
*Critical Thermal Minima (CT*_*min*_*)* **vs Body mass**		
All species	-0.2256	0.0830
*M*. *opacus*	0.3419	0.1401
*C*. *elphenor*	-0.1307	0.5828
*S*. *zambesianus*	0.0840	0.7248
*Chill Coma Recovery Time (CCRT)* **vs Body mass**		
All species	0.2936	0.0280
*M*. *opacus*	0.2171	0.3580
*C*. *elphenor*	-0.2905	0.2140
*S*. *zambesianus*	0.0395	0.9136

**Table 2:** The relationship between traits of high and low temperature tolerance and body water content (BWC). Analysis was performed using linear regression in STATISTICA. Dung beetle species BWC were correlated with traits of temperature tolerance independently and then all species combined. *R* represents the coefficient of correlation.

**Traits of high temperature**	**r**	***p***
Critical Thermal Maxima (CT_max_)		
All species	0.2593	0.00005
*M*. *opacus*	0.2879	0.0096
*C*. *elphenor*	-0.3109	0.005
*S*. *zambesianus*	-0.1793	0.1114
Heat Knockdown Time (HKDT)		
All species	0.4843	0.0000
*M*. *opacus*	0.4746	0.00001
*C*. *elphenor*	0.0504	0.6568
*S*. *zambesianus*	-0.1619	0.1515
**Traits of low temperature**		
Critical Thermal Minima (CT_min_)		
All species	0.0279	0.6670
*M*. *opacus*	0.3892	0.0004
*C*. *elphenor*	-0.2034	0.0703
*S*. *zambesianus*	-0.0445	0.6953
Chill Coma Recovery Time (CCRT)		
All species	0.5157	0.0000
*M*. *opacus*	0.3608	0.0010
*C*. *elphenor*	0.2023	0.0719
*S*. *zambesianus*	-0.0740	0.5143

**Table 3:** The relationship between body lipid content and traits of high (CT_max_) and low (CT_min_). Analyses were performed using linear regression in STATISTICA. Species body mass (*M*. *opacus* and *C*. *elphenor*) were correlated with traits of temperature tolerance independently and then all species combined.

Traits of temperature	r	*p*
Critical Thermal Maxima (CT_max_) vs Lipid content All species	0.2108	0.1916
*M*. *opacus*	0.1022	0.6681
*C*. *elphenor*	-0.2264	0.3372
Critical Thermal Minima (CT_min_) vs Body water content		
All species	0.2940	0.0656
*M*. *opacus*	0.6385	0.0024
*C*. *elphenor*	-0.4466	0.0484

### Effects of thermal variability on low temperature plasticity

**Fig 3:**
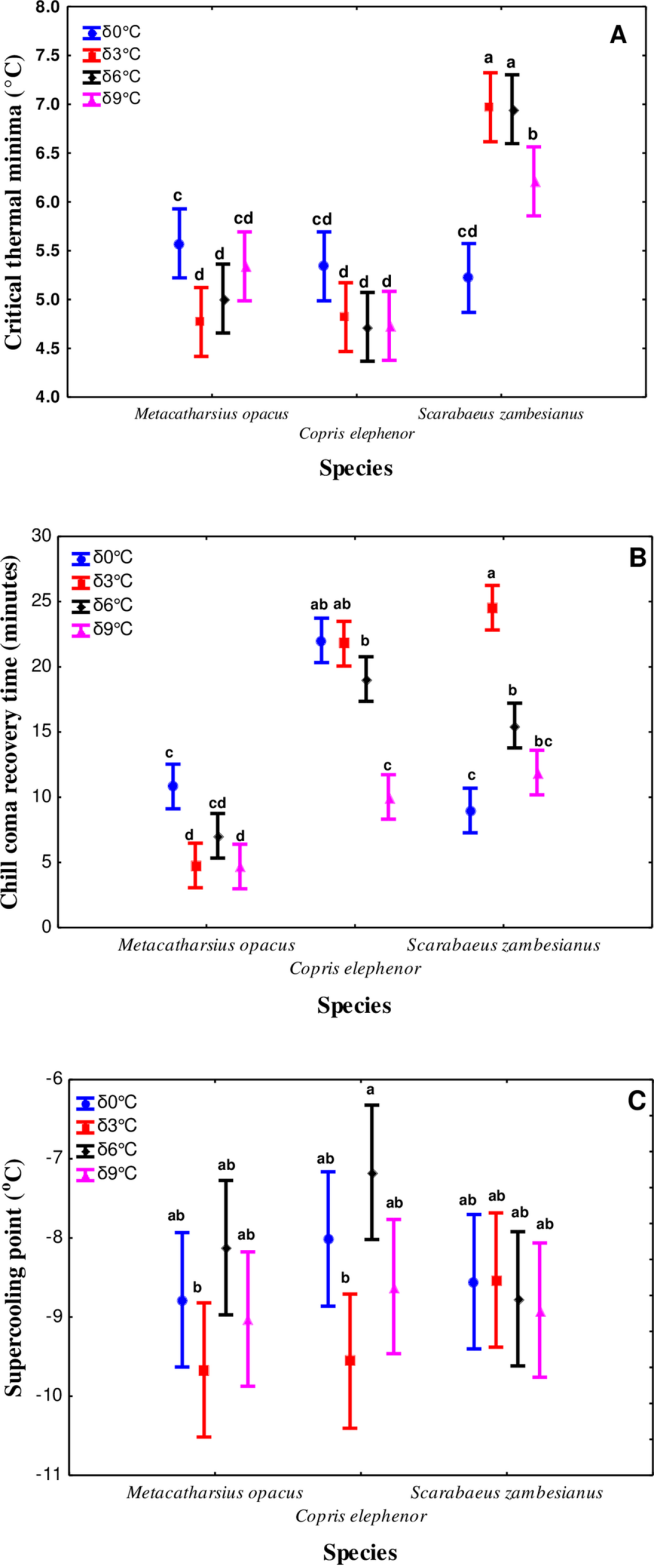
Effects of temperature fluctuations (acclimation at temperatures pulsating around a benign of 27°C: 0 = δ0°C (unacclimated), 3 = δ3°C, 6 = δ6°C and 9 = δ9°C) on (A) Critical thermal minima, (B) Chill coma recovery time and, (C) Supercooling points for *M*. *opacus*, *C*. *elphenor* and *S*. *zambesianus*. Vertical bars denote ±95% confidence limits. Means with the same letter are not statistically different.

Thermal variability had no significant relationships with traits of low temperature tolerance including CT_min_ (F_3,0.65_ = 1.25, *P* > 0.05). However, it had a significant relationship with CCRT (*χ*^2^ = 135.7, d.f = 3, *P* < 0.001) and SCPs (*χ*^2^ = 13.6, d.f = 3, *P* < 0.001). Nevertheless, higher thermal amplitudes significantly compromised CT_min_ relative to the control (δ0°C), a trend most apparent in *S*. *zambezianus* (Fig 3A).

In contrast, higher thermal fluctuations improved CCRT in both *M*. *opacus* and *C*. *elphenor*, unlike in *S*. *zambezianus* (Fig 3B). Similarly, SCPs were significantly enhanced by increased thermal amplitude in both *M*. *opacus* and *C*. *elphenor*, a trend not apparent in *S*. *zambezianus* (Fig 3C). Furthermore, species appeared to play a significant effect on CT_min_ (F_2,72_ = 92.9, *P* < 0.001) and CCRT (*χ*^2^ = 370.5 d.f = 2, *P* < 0.001) but not SCPs (*χ*^2^ = 3.48, d.f = 2, *P* > 0.05). *Scarabaeus zambezianus* had the most compromised CT_min_ compared to the other beetle species *M*. *opacus* and *C*. *elphenor*, which were not different from each other (Fig 3A). *Metacatharsius opacus* had a significantly shorter recovery time than the other beetle species following chill coma (Fig 3B).

Overall, body mass had contrasting relationships with low temperature tolerance (Table 1). Within species, body mass was not significantly related to CCRT, unlike when all species were pooled together (Table 1). Similarly, the relationship between BWC and low temperature tolerance was less pronounced, showing only significant correlation between CCRT and BWC when all species were combined, and a significant correlation for *M*. *opacus* for both CT_min_ and CCRT. As in high temperature versus BLC correlations, the relationship between BLC and low temperature tolerance was only undertaken for *M*. *opacus* and *C*. *elphenor*. Nevertheless, BLC had a significant relationship with low temperature tolerance (measured as CT_min_) for both *M*. *opacus* and *C*. *elphenor* (Table 3).

**Fig 4:**
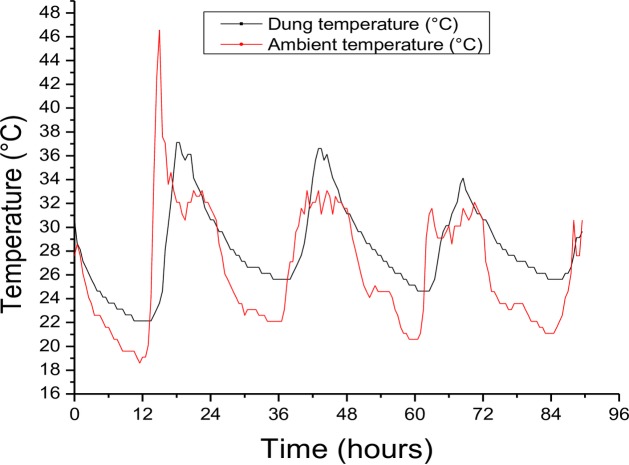
Differences in microclimate temperature records between ambient and ‘inside-dung’ during a one-week dung pat decomposition period in Khumaga village. Temperature was recorded using i-Buttons (DS1923, Maxim, Sunnyvale, CA, USA) at 15 minutes sampling interval for 5 days (January 19–23 2017). One i-Button was immersed in the middle of a freshly dropped cow dung pat, while the other one was placed in a shaded environment, ~1 m above the ground.

Microclimate temperature recordings revealed largely similar thermal conditions within the dung and the ambient environment (±2°C) with a few exceptions were dung temperature was higher (Fig 4).

## Discussion

Here the effects of exposure to thermal variability are reported for natural field-derived populations of coprophagic beetles. Our results show that higher amplitudes generally constrain the thermal fitness traits tested, with a few exceptions, highlighting the complexity of insect responses to temperature means and variability. Notably, CT_max_ and HKDT results generally showed that increased variability in temperature may compromise high temperature tolerance. This suggests that, under projected increase in thermal variability, the coprophagic species studied here may be at more risk of high temperature mortality, a trend that was apparent in *M*. *opacus* and *C*. *elphenor* (for CT_max_) and *S*. *zambezianus* for HKDT (Fig 2A and 2B). These results are consistent with findings by Terblanche et al [7] who demonstrated loss of basal and plastic responses to temperature in response to increased amplitude of temperature fluctuations.

Given that global climate change predictions suggest an increased frequency and variability of stressful temperatures [78,79], understanding the effects of these changes to insect population dynamics is important [80]. Related studies have looked at the effects of thermal amplitude on growth [81], fertility [82], density dependent net reproductive rate [57], development rate [83], stress resistance [84] and vector borne disease transmission [85]. However, none has considered natural populations of coprophagic species, with a view of understanding effects of climate variability on their activity and provision of ecosystem services. In addition, more recent studies have focused on how magnitudes of the variation impact species thermal performance curves [47,49, reviewed in 80]. Our current findings are therefore the first to show that increased amplitudes of thermal variations, typical of those predicted under global climate change, may have significant fitness costs to coprophagic beetles. This is likely to affect their provision of important ecosystem services [86,65].

Some studies suggest temperature variability may independently affect fitness traits, owing to the non-linearity of thermal reaction norms or thermal performance curves (see [56,70]). Our results also show that, while thermal amplitude play a significant role in fitness, species also differ significantly in their response to temperature. Among the three species, *S*. *zambezianus* appeared more tolerant to high temperature basally, compared to the other two beetle species. This further supports the idea that arthropod responses to climate variability are species-specific, and the rate of anthropogenic climate change may exceed that which may be compensated through genetic and evolutionary plastic mechanisms among the three different beetle species (e.g. [7]). The findings also suggest that *M*. *opacus* may be at more risk of high temperature mortality following higher thermal fluctuations compared to *C*. *elphenor* and *S*. *zambezianus*. Previous studies have also documented that that while plasticity to high temperature tolerance is an evident and near-ubiquitous feature of insect thermal physiology [9,87], the benefits may not be adequate to provide complete compensation for global climate change [88,87]. This therefore suggests that other mechanisms for compensation, e.g. behavioural modification and evolution may offer more benefit. However, it seems likely that many species may not be able to use behaviour e.g. tracking preferred microenvironments in space under prolonged environmental stress (e.g. [89]). This notion appears true for the dung beetle species tested here since ambient and dung temperature records were similar (Fig 4). It has been suggested that telocoprid dung beetle species (e.g. *S*. *zambezianus* studied here) withstand high temperatures by frequently mounting the dung to avoid excessive ground heat (of up to 60°C) during the day [90]. However, since our ground temperature recordings closely matched that of the dung it is unlikely that this ball-rolling behaviour would protect the species that were tested here from excessive heat. Furthermore, our study included two paracoprid species *C*. *elphenor* and *M*. *opacus*. The microclimate climate data recorded here suggest that physiological mechanisms to mitigate excessive heat are more important for their survival and preservation of key activities. However, the species tested here are nocturnal and perhaps gain significant performance and survival advantage due to cooler temperatures at night than day. It is possible that this nocturnal foraging behavior might be an evolutionary response to evade excessive day temperatures.

The correlation between body mass and high temperature tolerance was trait and species dependant. For example, in *S*. *zambezianus*, CT_max_ was positively correlated with body mass whilst HKDT was negative. The current results therefore contradict previous study by Nyamukondiwa and Terblanche [41] which found no evidence for the body mass constraints on thermal tolerance. However, our study supports studies where body mass was shown to correlate with heat tolerance [40,45,91]. We also found that increased heat tolerance was associated with increased BWC, as previously reported [45], perhaps due to enhanced resistance to desiccation [92]. This may be because insect mortality at high temperatures is closely associated with rapid water loss leading to desiccation [9,92]. Thus, insects with higher BWC may take more time to desiccate, and may have improved survival at high temperature through the link between the two stressors (see [93]). We did not find any correlation between traits of high temperature and BLC for the beetle species tested here, indicating that body lipid content may not play a role in high temperature tolerance.

Thermal variability had more dramatic effects on CCRT compared to CT_min_ and SCPs. The results therefore highlight differences in sensitivity to thermal variability among related traits of thermal tolerance. For *S*. *zambezianus* CT_min_, higher thermal amplitudes also compromised low temperature tolerance (Fig 3A). It is also interesting to note that *S*. *zambezianus* had the highest lower activity temperature limit (CT_min_), which perhaps relates to its superior high temperature tolerance compared to the other species (see Fig 2A and 2B), since this may have come at a cost of fitness at low temperature (CT_min_) [94]. Indeed, similar fitness costs have been reported in other insect taxa, [95–97]. Similar modest thermal fluctuations have been observed to affect many insect life history traits including mating behaviour and fertility (see [9]). Similarly, Marshall and Sinclair [98] showed that at stressful low temperatures, *D*. *melanogaster* trades off immediate survival for future reproductive output. Thus, thermal variability reported here may also have significant life history trade-offs for the beetle species tested. Coupled with individual reduced fitness, thermal variability under changing climates may therefore have far reaching population level consequences [47]. Nevertheless, exact trade-offs resulting from the magnitude of the thermal variability in *M*. *opacus*, *C*. *elphenor* and *S*. *zambezianus* warrants future investigation.

Previous studies have indicated that ectotherms are already experiencing sub-lethal low temperatures, e.g. chill coma and CT_min_ in natural environments [47], and consistently compromise fitness traits under increased magnitude of temperature changes [9,70]. While the direct and indirect effects of increased thermal variability have been appreciated by evolutionary ecologists [56,70], mechanistic models for surviving variable stressful environments have been limited. These mechanisms however remain significant in explaining how organisms likely cope with imminent and increased thermal variability associated with climate change. Without compensatory physiological or behavioural modifications to fluctuating thermal regimes, current findings thus suggest fitness losses for the beetle species tested here. Measurement of traits of low temperature *vis* CT_min_, CCRT and chill coma also reveal significant patterns of species local low temperature adaptation and compensatory responses under thermal variability (see [9]). However, Donat and Alexander [99] suggest cold events may be less likely as opposed to extreme high temperature events under climate change. Thus survival of species under variable high temperature may be more imminent as opposed to low temperatures. Nevertheless, while low temperature variability may not be that critical here, it has shifted key life history traits, seasonal timing, and population phenologies of many insect species [100].

Body mass generally had little impact on low temperature tolerance traits (Table 1) even when traits for all species were pooled together, in particular for CCRT. This result corroborates with Nyamukondiwa and Terblanche [41] who showed no effects of body mass on insect CTLs to activity. Similarly, the relationship between high temperature tolerance and BWC did not follow a specific pattern. For example, BWC appeared to be positively correlated with CT_min_ and CCRT for *M*. *opacus*, and when species were pooled together for CCRT. This suggests taxa and trait dependent effects of varying thermal amplitudes to low temperature tolerance. Our results also showed significant correlations between traits of low temperature and BLC. Indeed, [101] elaborated on the role of metabolites, including carbohydrates (i.e. trehalose), free amino acids, lipids, osmoprotectants and polyols are upregulated during low temperature conditions. Our results therefore suggest lipid accumulation may be an essential mechanism sustaining low temperature survival by insects faced with increased magnitude of low temperature fluctuations.

Age and body condition e.g. nutrition are among some of the factors which may affect physiological and biochemical processes in insects, including traits of thermal tolerance measured here [9,34,38]. Thus, one confounding effect in our study here is that, age and nutritional history of the test organisms was not strictly controlled for. Nevertheless, our assessments of body mass, BWC and BLC and their correlation with thermal tolerance traits may be indicative of body condition which can correlate with age. In conclusion our results show that coprophagic insects *M*. *opacus*, *C*. *elphenor* and *S*. *zambezianus* may suffer fitness losses (for both high and low temperature traits) in response to increased temperature variability with climate change. Tolerance levels varied significantly across variability (δ) levels, and likely depend on the trait in question and the species. Field studies have reported that pesticide residues in the dung of treated animals may have deleterious consequences on different developmental stages of dung beetles [102,103]. Thus loss of fitness with thermal variability reported here, coupled with reports on the effects of pesticides (e.g. [103]), represent a significant burden to conservation of dung beetle ecological services.
